# Elephant (*Elephas maximus*) temporal activity, distribution, and habitat use patterns on the tiger’s forgotten trails across the seasonally dry, subtropical, hilly Churia forests of Nepal

**DOI:** 10.1371/journal.pone.0216504

**Published:** 2019-05-13

**Authors:** Kanchan Thapa, Marcella J. Kelly, Narendra Man Babu Pradhan

**Affiliations:** 1 WWF Nepal, Baluwatar, Kathmandu, Nepal; 2 Department of Fish and Wildlife Conservation, Virginia Tech, Blacksburg, VA, United States of America; 3 International Union for Conservation of Nature, Lalitpur, Nepal; University of South Carolina, UNITED STATES

## Abstract

Understanding spatial distribution, habitat use, and temporal activity patterns is important for species conservation planning. This information especially is crucial for mega herbivores like elephants as their ranging patterns encompass a myriad of habitats types. Churia habitat is geological fragile yet important for wildlife in Nepal and India. We used camera trapping and sign surveys covering 536 km^2^ of Churia and surrounding areas within Chitwan National Park. Across 152 trapping locations, we accumulated 2,097 trap nights in a 60-day survey during the winter season of 2010–11. We used a non-parametric kernel density function to analyze winter activity patterns of elephants detected in camera-traps. Additionally, we walked 643 km over 76 grid cells in two surveys (winter and summer) to estimate elephant distribution and intensity of habitat use using an occupancy framework. Multi-season models allowed us to make seasonal (winter versus summer) inferences regarding changes in habitat use based on covariates influencing use and detection. We photographed 25 mammalian species including elephants with calves with a trapping rate of 2.72 elephant photos events per 100 trap nights. Elephant winter activity pattern was found to be mainly nocturnal, with crepuscular peaks. Covariates such as normalized differential vegetation index and terrain ruggedness positively influenced elephant spatial distribution and habitat use patterns within the Churia habitat. We also found lower elephant habitat use ($\hat{\Psi }SE(\hat{\Psi })$) of Churia in winter 0.51 (0.02) than in summer 0.57 (0.02). Elephants heavily used the eastern portion of Churia in both seasons (67–69%). Overall, Churia habitat, which is often ignored, clearly is used by elephants, with increases in summer use in the west and high use year-round in the east, and thus should no longer be neglected or forgotten in species conservation planning.

## Introduction

Distribution of resource availability and habitat use by animals within a complex and dynamic landscape is a central theme in conservation ecology [1]. Understanding distribution and abundance of wildlife species is critical for setting appropriate wildlife management goals, monitoring effectiveness of management interventions, and informing policy makers, the general public, and other stakeholders. Understanding resource distribution and use is crucial especially for conservation of wide ranging, yet endangered, species like Asian elephants (*Elephus maximus*) with variable home range sizes from 30 km^2^ to > 600 km^2^.

**Fig 1:**
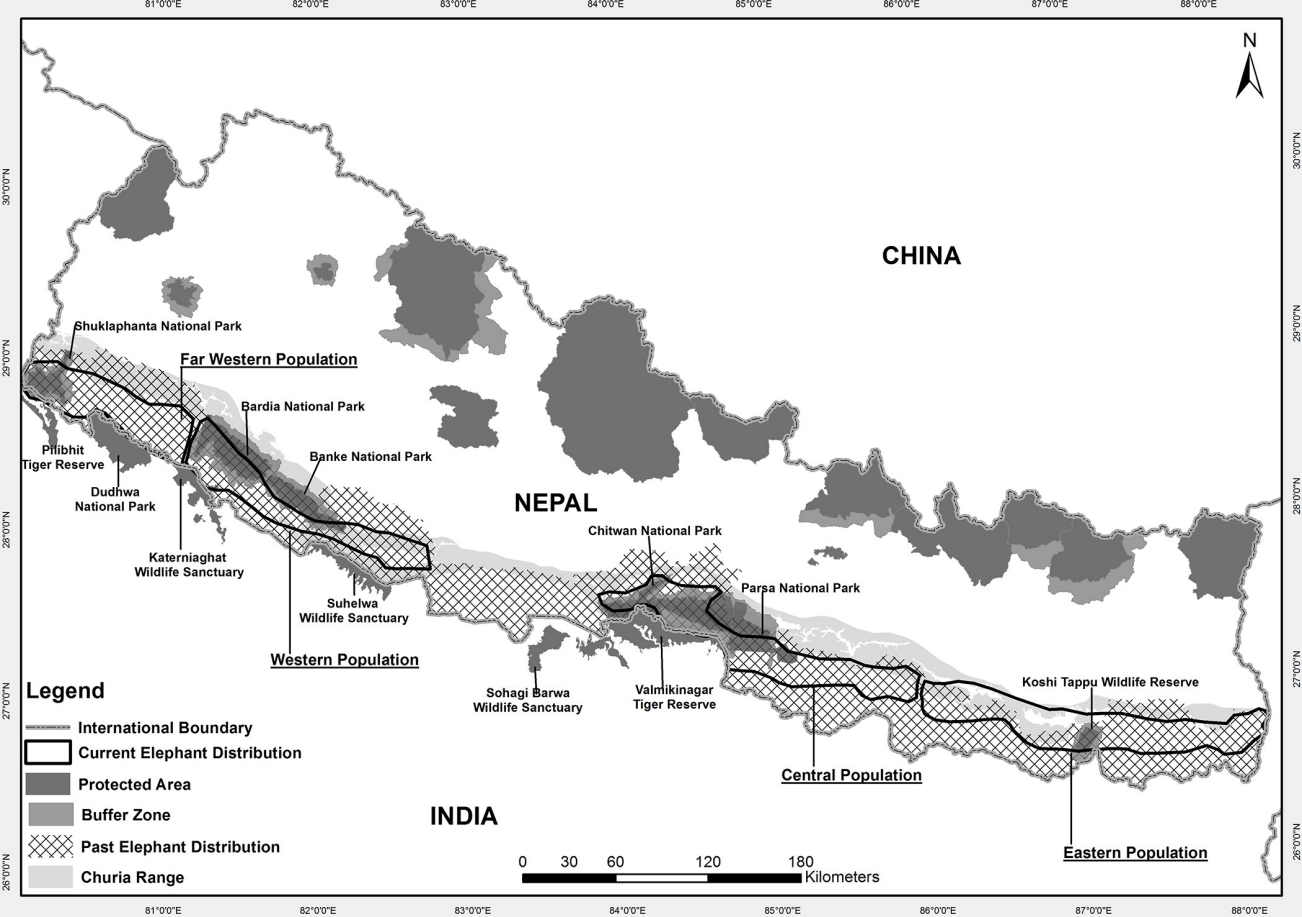
Past and current distributional range of Asian elephants and the Churia habitat in Nepal (a.k.a. Siwaliks in India).

The Asian elephant was once distributed from the Tigris-Euphrates in west Asia eastward into the Indian subcontinent, to South and South East Asia, and to Yunnan Province in China. Today, the range of the Asian elephant is confined to the Asian continent, but elephants are distributed discontinuously across this range. Elephants in South Asia, which receive far less attention than African elephants, *Loxodonta africana*, persist today in small, insular populations restricted largely to protected areas (PAs) within fragmented landscapes. In the early nineteenth century, elephants occurred across the entire lowland of Nepal [2] but now, wild populations of elephants exist in four possible sub-populations spread in pockets along low lying areas of Nepal (Fig 1). A recent study shows that 3,365 km^2^ of intact forest in the central sub-population, forming what is known as the Chitwan-Parsa-Valmiki Complex, contains approximately 20–25 elephants. The hilly Churia range forms a significant part (~36%) of this complex and these forest blocks provide connectivity between PAs within the complex (Fig 1). Four management units for Asian elephants have been proposed in the Indian peninsula [3], where the northeastern population has been defined as a single management unit. The Churia range is believed to provide connection across a significant part of this northeastern elephant range.

The Churia, also called the Siwaliks in India, is one of the youngest of the five mountain ranges in Nepal [4] extending from the Brahmaputra River in the East in India to the Indus River in the west in Pakistan [5] and occupies 13% of the country’s total land surface [6]. Forest density within the Churia is high (73% has intact forest cover) and conservation of Churia is critical to maintain trans-border connectivity across the landscape in Nepal and India [7, 8].

Along the foothills of the Himalaya, past classic studies on elephant ecology have focused on the lowland areas, which comprises of alluvial floodplain grasslands, riverine forests, and climax *Shorea robusta* forests [9, 10]. At the landscape level, the only data on elephant habitat use comes from Lamichhane et al. [11], using a combination of sign and questionnaire surveys across the lowland area of Nepal, but data were not segregated based on habitat type. Moreover, habitat use and/or preferences and activity patterns are poorly known for elephants from seasonally dry deciduous forests in the Churia hills. This lack of knowledge is particularly worrying for conservationists given the pervasive threats of habitat loss and over exploitation of elephant populations [12]. Additionally, the Churia of Nepal generally suffers from degradation and over-exploitation via agricultural encroachment and poaching [8, 13].

Thus, if an elephant population exists within the Churia forests, with 639 km^2^ of habitat in CNP alone and a total of 1,921 km^2^ of this physiographic zone, the hilly terrain could represent high potential for elephant conservation. To date, however, there have been no studies examining intensity of habitat use or activity patterns of these large pachyderms in Churia forest habitat. Thus, habitat and site-specific assessments are needed to make better informed conservation management decisions for these endangered species [14] in Churia habitat.

We used a combination of methods including camera trapping to estimate elephant trapping rates and temporal activity patterns, and sign surveys to examine factors influencing the distribution of elephants, intensity of habitat use, and seasonal changes in habitat use in the relatively unknown Churia forest hills of Nepal. We collected camera trap data and sign data for a study originally designed for tigers in an area that was relatively unstudied[8]. Thus, these “forgotten trails” within Churia are an important source of data acquisition for elephants, which have similar space requirements as tigers.” We employed an occupancy modeling framework [15] that relies on spatial and temporal (season) replication [16] and uses elephant sign surveys to investigate the distribution and habitat use patterns of forest elephants [7].

## Materials and methods

### Ethics statement

The study was conducted within in Chitwan National Park, Nepal after gathering necessary research permits from Department of National Park and Wildlife Conservation. We used non-invasive method such as camera trapping and recording indirect signs left by animals, thus animal care and use committee approval was not required.

### Study area

**Fig 2:**
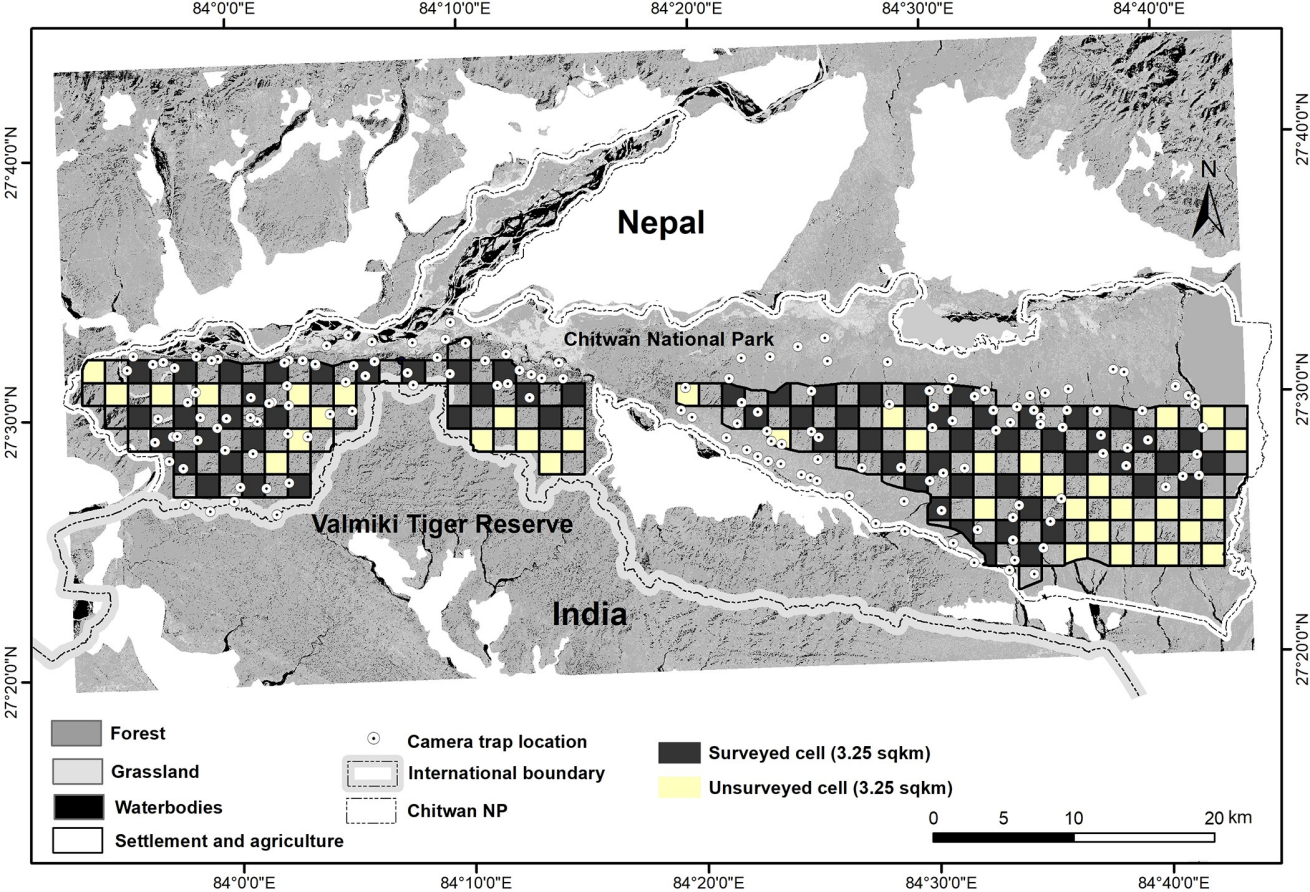
Study area showing Churia habitat within Chitwan National Park, Nepal. A total of 152 camera trap locations were used and 76 grid cells each measuring 3.25 km^2^ were surveyed for elephant sign to determine spatial and temporal patterns in habitat use of elephants using an occupancy framework in seasonally dry sub-tropical habitat in year 2010/11.

We focused efforts in the Churia habitat of Chitwan National Park (CNP), a 639 km^2^ subset of the total park (area km^2^) located in central Nepal (Fig 2). The hilly Churia habitat stretches between the flat lowland areas on the eastern side (363 km^2^) and between the lowland forest of Chitwan National Park and Valmiki Tiger Reserve on the western side (276 km^2^). Churia habitat is contiguous with the southern buffer zone to form the Madi Valley with a high human population density of 440 per km^2^ [8]. Churia habitat forms the main interlinking hill forest block that provides connectivity to Valmiki Tiger Reserve in India, and the Parsa National Park and Chitwan National Park in Nepal, to form a Chitwan-Parsa-Valmiki protected area complex [17].

Churia habitat is an undulating, hilly terrain with elevation ranging from 150 m to 714 m. The ecosystem is dynamic in nature, with fragile substrate conditions. The top soil layer is very thin with stones and boulders beneath it. Deciduous forest trees shed their leaves at the onset of winter season and the forest are prone to fire during summer season. Most of the ground vegetation and forest floor is cleared by natural forest fires in the summer. Seasonal and perennial rivers originate in the Churia and cascade down to the lowlands to form important sources of the water for wildlife and people living in the lowland Terai. Churia habitat is composed of mixed deciduous forest with mixed pine (*Pinus roxburgii*) forests emerging at ~400 m elevation and upwards. Broom grass *Thysanolaena maxima*, asparagus *Asparagus officinalis*, and date palm *Phoenix dactylifera* are the main non-timber forest products (NTFP), which are illegally harvested and traded in the local markets.

Major herbivores in the Churia include gaur, sambar, barking deer, chital, wild pig, and primates; supporting a density of 62.5 animals/km^2^ for small to large sized prey species combined [8]. The steep slopes of the Churia support four species of antelope: the Himalayan serow (*Capricornis thar*), the goral (*Naemorhedus goral*), the four-horned antelope (*Tetracerus quadricornis*) and the nilgai (*Boselaphus tragocamelus*). Further details on the Churia habitat in Chitwan National Park are described elsewhere [7, 8, 18].

### Camera trapping survey

We conducted a single camera trap survey [8] in the winter season from December 2010 to March 2011. We sampled 576 km^2^ of Churia habitat and divided the study area into four survey blocks each measuring on average 132 km^2^ (SE 23.70). Each of the blocks was divided further into 2 X 2 km grid cells and we deployed camera stations within each cell (Fig 2). We set pairs of cameras at 152 locations based primarily on accessibility, with an average of 40 (SE: 3.0) camera stations per block. We used combinations of camera trap models—Moultrie D50, Moultrie D55, and Bushnell model. The inter-trap distance between two consecutive locations ranged from a minimum of 600 m to maximum of 3,519 m with average distance of 1.56 km (SE 0.09). Due to limited number of cameras, we followed the fourth design protocol [19] with rotation of camera traps from block to block sequentially to cover the area of interest. We placed camera traps on river banks (dry and wet, n = 119), animal and human trails (n = 29), and fire-lines (n = 10). The survey was originally designed to maximize capture probabilities of tigers in the habitat, however camera traps also obtained substantial numbers of photographs of large herbivores such as elephants, which also use similar travel routes as tigers [20].

### Sign survey

We conducted two sign surveys, one in the winter (December-March) and one in the summer (April-June) season. For animals with larger home ranges than our 3.24 km^2^ grid cells, such as elephants, occupancy models yield reliable estimates of probability of detection and habitat-use (rather than true occupancy) at finer spatial scales [21]. Within the home range, occupancy can be seen as a metric of intensity of habitat use, which has been successfully used in earlier studies for tigers [7, 22], dholes [23], and elephants [24]. We created a grid across the Churia with each grid cell (n = 104) of size 3.24 km^2^ as spatial sampling units for measuring intensity of habitat use and detection probabilities in Churia habitat (Fig 2). Our objective was focused on how forest elephants use habitat within a narrow stretch of Churia, assuming that in a hilly environment, 3.25 km^2^ provides adequate space for foraging. Elephant signs (e.g. tracks, scrapes, dung, urine, etc.) were collected at grid cell level. We surveyed 76 grid cells (out of 104 possible) in a checkerboard pattern (Fig 2) sampling every other cell using 8 spatial replicates; each replicate consisting of a 600 m transect. Total survey effort within each 3.24 km^2^ grid cell was 4.8 km. We walked transects and recorded field observations along every 600 m and considered these 600 m stretches as spatial replicates (i.e. encounter occasions in occupancy models). Observations recorded across each 600 m were either termed as “0” for non-detection or “1” for detection of elephant sign.

We used landscape level covariates to determine the probability of elephant habitat use across the grid cells. These covariates included a terrain parameter: terrain ruggedness index (TRI, [25]) computed from a digital elevation model (DEM) with-90m resolution data. Remotely sensed vegetation indices have been used as potential tools in investigating distribution and habitat relationships [26] for large herbivores such as elephants [24]. Thus, we used these variables: a) index of vegetation characteristics that indicates the amount of primary productivity: Normalized Difference Vegetation Index (NDVI) extracted for winter; b) variables characterizing available habitat (HAB) estimated as the proportion of sal (*Shorea robusta*) dominant mixed deciduous forest within each grid cell. and c) tree canopy cover (CC). We also used distance to nearest settlement (DNS) extracted as a surrogate measure of disturbance at the landscape level [7]. All variables were extracted from GIS public domain, and values were averaged at the grid cell level. We expected elephant intensity of habitat use to be positively influenced by available habitat, NDVI, canopy cover, distance away from settlement, and negatively influenced by terrain ruggedness. We also used a multi-season occupancy framework to determine if there were seasonal differences in winter versus summer in detection probability and habitat use as substrate conditions change between season. Detection probability is expected to be lower in summer than in winter, due to increased vegetative thickness and substrate condition, but increased vegetative thickness may cause elephants to increase their use of such as habitat due to increased forage. Thus, we expected seasonal differences in elephant detection and habitat use (see Table 1 for more details).

**Table 1:** Field level and landscape level predictor variables (including their justification) evaluated as covariates affecting elephant habitat use in the Churia habitat. The “+” and “-” indicate positive versus negative apriori predictions regarding the hypothesized direction of the effect of the covariate on habitat use by Asian elephants.

Covariate	General justification for the selection of the covariates	Description	Value Range			Hypothesized *Apriori* relationship to habitat use probability
			Min	Max	Av. (SD)	
**Terrain Ruggedness Index** **(TRI)**	Churia is hilly region and elephant in general tends to avoid area with high TRI.	Computed using the SRTM digital elevation model-90m [35]	71.31	276.48	175.69 (46.51)	**-**
**Habitat Available (HAB)** **(km**^**2**^**)**	Habitat availability is the amount of total habitat available in the area. Higher the availability higher elephant habitat use in the region [27]	Derived and extracted for the study area from 2010 supervised classification Landsat 6 Thematic Mapper imagery (28.5 m X 28.5 m resolution) with permission from WWF Nepal. Download from: http://Glovis.usgs.gov	0.08	2.64	2.52 (0.39)	+
**Canopy Cover** **(CC)** **(km**^**2**^**)**	Canopy cover is important as it provides the shade and dictates the kind of ground cover [32]. Higher the canopy cover, higher elephant use in Churia.	Derived and extracted for the study area from 2011 supervised classification of Landsat 4–5 Thematic Mapper imagery (30 m X 30 m resolution) with permission from WWF Nepal.	0	2.56	1.98 (0.56)	+
**Normalized Difference Vegetation Index (NDVI)**	NDVI has been used as the used as a measure of vegetation primary productivity [26]. Elephant habitat tends to increase with increase in vegetation productivity (NDVI)	Derived from Landsat 6 Thematic Mapper imagery (28.5 m X 28.5 m resolution) of the study area during the ‘winter season’ (November 2011). Download from: http://Glovis.usgs.gov	0.19	0.50	0.43 (0.05)	+
**Distance to Nearest Settlement** **(DNS)**	Elephant habitat use are higher in the core area tends to increase with distance from the settlement areas (proxy to disturbance) [28]	Generated a surface by calculating the Euclidean distance from settlement data extracted from Nepal Survey Department 1996 digital topographic data and world settlement data	0	11	5.05 (2.6)	+

Min: Minimum; Max: Maximum; Av.: Average; SD: Standard Deviation

### Data analysis

#### Trap rates

We sorted all the camera trap pictures and considered photos as independent events if they were 30 minutes or more apart, unless we could tell there were distinctly different individuals, as is commonly done in camera trap studies [29, 30]. We found it difficult to individually identify elephants, thus we used capture events (number of independent photos) per unit effort (per 100 trap nights) to measure the trapping rate of elephants [31, 32].

#### Temporal activity pattern

We used a non-parametric kernel density function to analyze temporal activity patterns [33] of elephants detected in camera-traps in our winter survey. The time stamp of each independent detection was used to fit the density function and analysis was conducted using ‘circular’ package in R [34].

#### Distribution and intensity of habitat use

We used the standard occupancy framework [15] to model elephant occupancy at the within home range scale, maximizing the likelihood of observing the detection history at the sites. We used single species, multi-season, correlated-detection occupancy models in Program PRESENCE [35], that explicitly consider spatial autocorrelation in detection in adjacent 600 m transects within each grid cell [16]. The Hines et al. [16] model also derives the probability of habitat use (initial) for the winter season, probability of habitat use (derived) in the second season and trend in habitat use over time (λ).

We fit a small set of ten candidate models meant to reflect hypotheses regarding the effects of covariates on habitat use (8 potential models) and detection processes (2 potential models). Before modeling, all covariates were screened for collinearity. Highly correlated variables (|r_s_| ≥ 0.77) were either removed or not used in combination within the same model. All covariates used in modelling were normalized using the z transformation and/or scaled using a constant value [22]. We used a two-stage approach to model the parameter of interest at the grid cell level [36]. In first stage, we modelled detection probability either as constant or varying by season while using a global model (model containing all the five covariates: TRI+DNV+NDVI+HAB+CC) influencing probability of habitat use. In the second stage, we fixed the top model for detection and built models using different combinations of covariates influencing habitat use, following the approach of Sunarto et al.[22]. For model selection, we ranked all models using Akaike’s Information Criterion corrected for small sample size (AICc) and chose the best model based on lowest AIC scores. We considered all models with ΔAIC_c_ < 2 as competing models [37]. We used model averaging techniques to determine the cell-specific probabilities of habitat use (Ѱ_CELL_) considering all the competing models. The value of untransformed coefficients (i.e. betas, β) reflects the magnitude and direction (sign) of the influence of covariates on the probabilities of habitat use. We considered the influence of covariates as important and supported if their β estimates and the 95% confidence limits did not include zero [38]. We reported the model averaged final estimates (as probability of habitat use) on the parameter of interest as well as the trend in habitat use between seasons (λ, winter to summer). A λ of <1.0 would indicate decrease in habitat use from winter to summer while λ of >1.0 would indicate increase in use. To estimate overall elephant probability of habitat use within the Churia habitat of Chitwan National Park, we weighed the cell-specific habitat use estimates by potential habitat within each grid cell (3.24 km^2^) [36]. The computation of variance was done using a parametric bootstrapping approach [36]. We prepared predicted maps of elephant habitat use based on inferences made from the model averaged outputs in ArcGIS (Version 10.1). We used relevant covariates from top models to predict elephant habitat use across the study site including the non-surveyed grid cells. We used the null model estimates as a proxy for dynamic occupancy over two-time frames, showing proportion of sites not used at time t that were used at time t_+1_(colonization–became used), and proportion of sites used at time t that were and not used in t_+1_(extinction–became unused).

## Results

### Elephant trap success and temporal activity pattern

**Fig 3:**
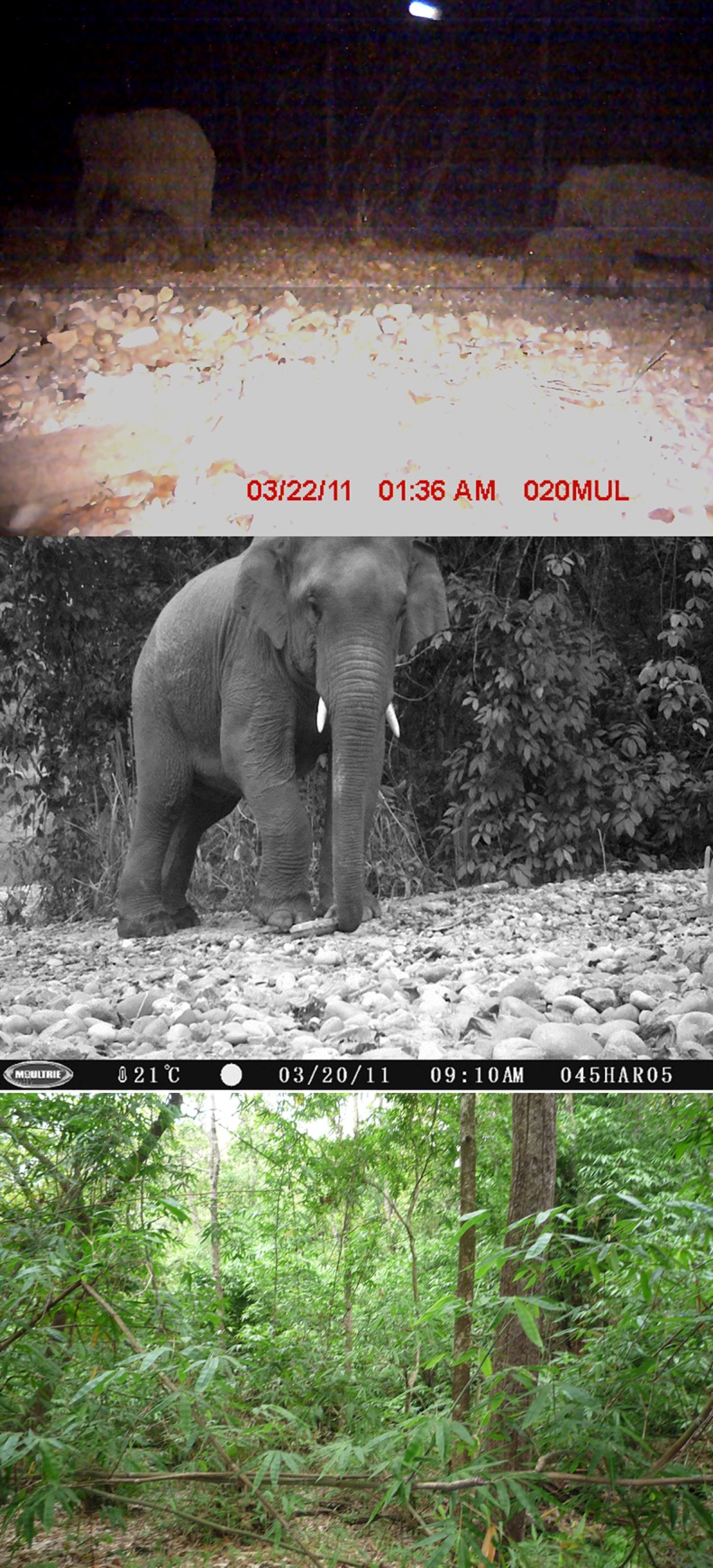
Camera trap images of elephants (including a calf) and bamboo patches used as forage by elephants in seasonally dry subtropical Churia habitat.

**Fig 4:**
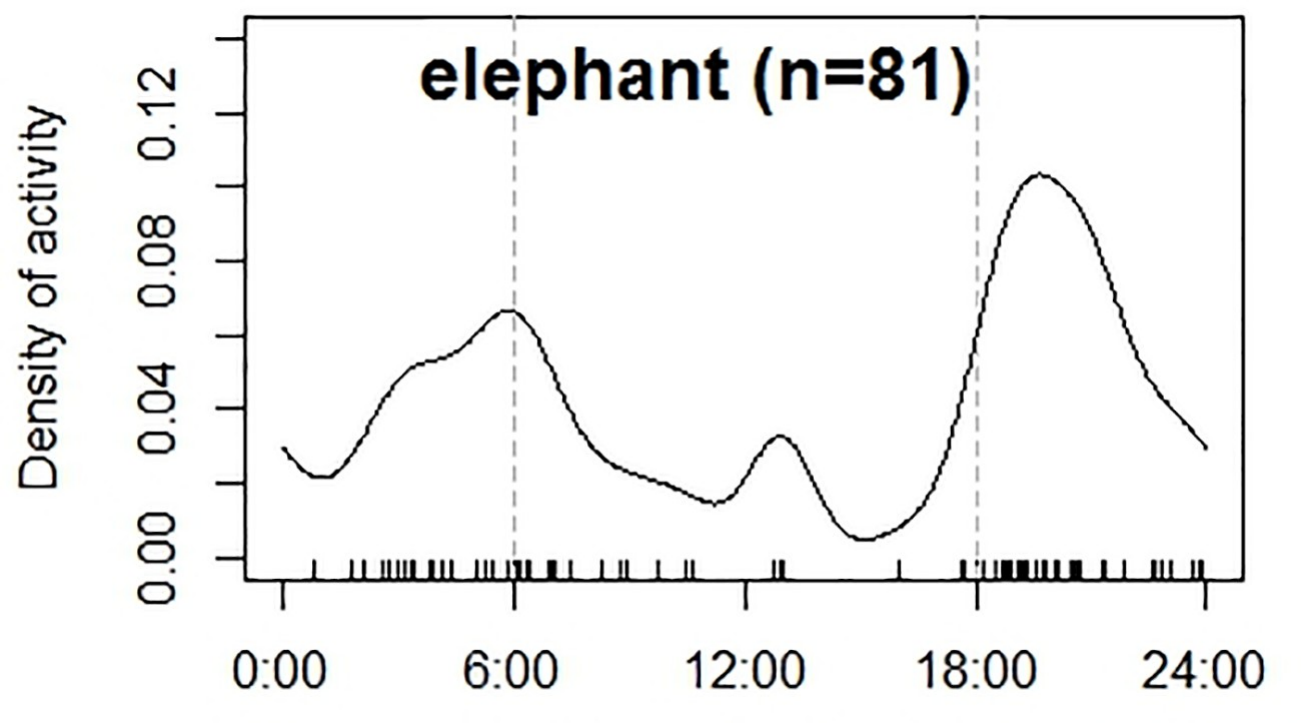
Temporal activity patterns of elephants in the winter season in Churia habitat, Nepal in year 2010/11. The continuous lines represent activity of elephants as detected in 81 independent events captured by camera traps. The vertical dotted lines represent timing of sunrise (~ 6:00 hrs.) and sunset (~18:00 hrs.) in the winter season.

We amassed 6,332 mammalian photographs in 2,097 trap nights after removing 123 trap nights of camera malfunctions. A total of 4% (~254 photographs) of these were elephant photos including a total of 81 independent events of these mega herbivores. The trapping rate was 2.72 independent photos of elephants per 100 trap nights. We located a minimum of three large herds of elephants in the Churia habitat of Chitwan National Park during the winter season (Fig 3). Two of these were potential breeding herds of elephants with two independent detections of two individual calves in separate locations within the same trapping block. We found elephants showing crepuscular activity in the Churia habitat in winter with activity peaking just after sunset (Time: 6:00 PM) and near sunrise (Time: 6:00 AM) in the Churia habitat (Fig 4).

### Elephant detectability and habitat use

**Fig 5:**
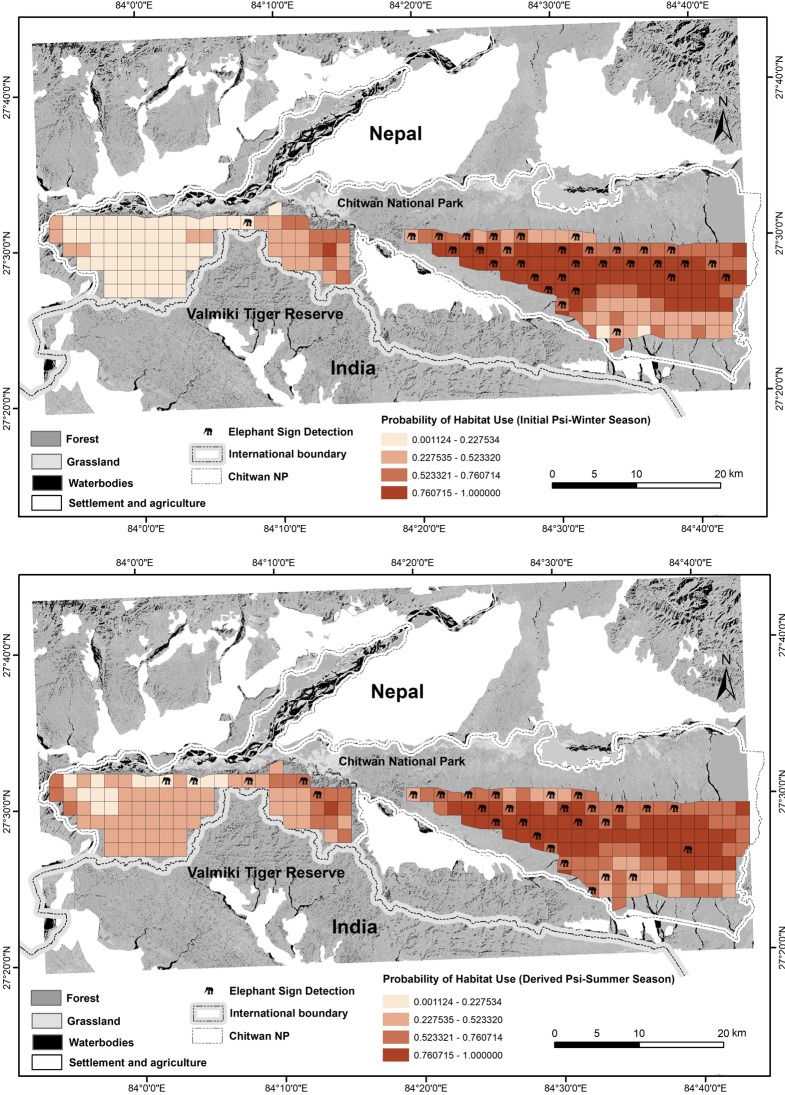
Patterns of spatial and temporal (season: winter (top); summer (bottom)) variation in probability of elephant habitat use in Churia habitat of Chitwan National Park, Nepal, based on occupancy modeling using the spatial autocorrelation model developed by Hines et al. [16].

During the sign survey, the team walked a total of 643 km of trails in the winter (331 km) and summer (312 km) seasons and detected a total of 362 fresh signs of elephants in 40 of the 76 grid cells in two seasons in the Churia habitat (Fig 5). No signs were detected in grid cells located in the western section of the Churia habitat, however elephant signs were recorded in a few grid cells surrounding the western Churia habitat.

We compared the standard MacKenzie et al.[39] occupancy model with the Hines et al. [16] model that explicitly addresses spatial auto-correlation of sign detections made along adjacent 600 m spatial replicates. Based on AIC values, the Hines et al. [16] models better fit the data than the standard model that assumes sign detections are independent (Table 2). Therefore, we used the Hines et al.[16] model incorporating spatial autocorrelation when including covariates.

**Table 2:** Comparisons between the standard multi-season occupancy model [39] and the multi-season model including potential auto-correlation of sign detection [16] used to estimate habitat use of elephants at the 3.24 km^2^ grid cell level (Ψ_CELL_). Surveys were conducted in winter (2010–2011) and summer (2011) seasons in the Churia habitat of Chitwan National Park, Nepal.

Model	AIC	ΔAIC	*w*_*i*_	Model likelihood	K
Ψ_CELL_, θ^0^(.), θ^1^(.),γ (.),ε(.),p(.), pi(.)	787.28	0.00	0.89	1.00	6
Ψ_CELL_, γ (.),ε(.),p(.)	791.49	4.21	0.11	0.1218	4

Ψ_CELL_ = probability of site occupancy/habitat use at the grid cell level; *p* = probability of detection; AIC is Akaike's information criterion, ΔAIC is the difference in AIC value of the focal model and the best AIC model in the set, K is the number of model parameters and –2Loglik is -2 of the logarithms of the likelihood function evaluated at the maximum. θ^0^ = spatial dependence parameter representing the probability that the species is present locally, given the species was not present in the previous spatial replicate; θ^1^ = spatial dependence parameter representing the probability that a species is present locally, given it was present at the previous spatial replicate. γ is the probability that the site is occupied in summer season, given that it was unoccupied in winter season. ε is the probability that the site is unoccupied in summer season, given that it was occupied in winter season. pi is the probability that initial replicate is preceded by an occupied site.

**Fig 6:**
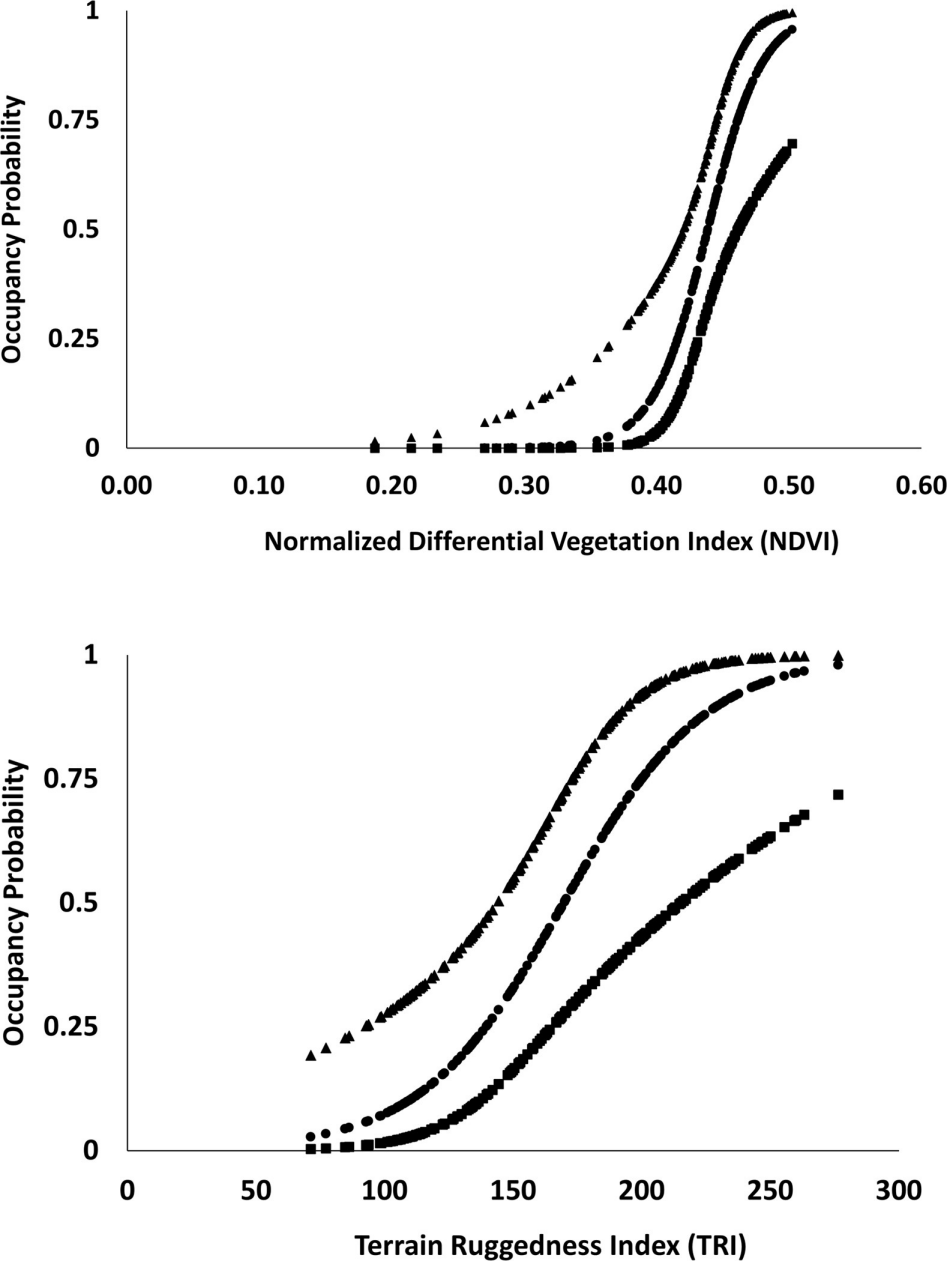
Relationships between the highly influential continuous covariates based on beta estimates (β) from top models and the probability of elephant habitat use in Churia habitat in Chitwan National Park, Nepal. “●” represent mean Psi, “▲” represent the “upper confidence limit”, “■” represent lower confidence limit.

At the grid cell level (i.e. 3.25 km^2^), the probability of detecting elephants, $\hat{p}(\mathrm{SE}(\hat{p}))$, was influenced by season with higher probability of detecting elephant sign along the 600 m transects in winter (0.89 (0.06)) than in summer at 0.69 (0.09) (Table 3). While there was a competing model without seasonal influence on detection, that model held only 33% of the model weight compared to 67% with seasonal effect. Therefore, we decided to use seasonal variation in detectability in the subsequent analyses for modelling probability of elephant habitat use. Among all five landscape covariates, additive effects of Churia productivity (NDVI) and terrain ruggedness index (TRI) were included in the top models influencing elephant habitat use (Table 4, Fig 6). Among the top models, NDVI had a high positive effect on elephant habitat use with CIs that did not overlap zero; while TRI also was found to be positive, but potentially inconclusive due high variability and CIs overlapping zero (Table 5).

**Table 3:** Top competing models for elephant detection probability (p), including the influence of covariate (season) on detection of elephant sign on 600 m transects in the Churia habitat of Chitwan National Park, Nepal based on habitat covariates. The global model represents additive effects of all the covariates used on occupancy.

Model	AIC	ΔAIC	*w*_*i*_	Model likelihood	K
Ψ_CELL_ (Global), θ^0^(.), θ^1^(.),γ (.),ε(.),p(season), θ^0^pi()	729.66	0.00	0.67	1.00	15
Ψ_CELL_ (Global), θ^0^(.), θ^1^(.),γ (.),ε(.),p(),θ^0^pi()	731.1	1.44	0.33	0.49	14

Ψ_CELL_ = probability of site occupancy/habitat use at the grid cell level; *p* = probability of detection; AIC is Akaike's information criterion, ΔAIC is the difference in AIC value of the focal model and the best AIC model in the set, K is the number of model parameters and –2Loglik is -2 of the logarithms of the likelihood function evaluated at the maximum. θ^0^ = spatial dependence parameter representing the probability that the species is present locally, given the species was not present in the previous spatial replicate; θ^1^ = spatial dependence parameter representing the probability that a species is present locally, given it was present at the previous spatial replicate. γ is the probability that the site is occupied in summer season, given that it was unoccupied in winter season. ε is the probability that the site is unoccupied in summer season, given that it was occupied in winter season. pi is the probability that initial replicate is preceded by an occupied site.

**Table 4:** Top models for elephant probability of habitat use (Ψ_CELL_), in 2010 and 2011, including the influence of covariates on habitat use in Churia habitat of Chitwan National Park, Nepal based on modelling (seasonal effect or not) and probability of detecting elephant sign *p* on 600 m transects. The final model in the table represents the null (constant model).

Model	AIC	ΔAIC	*w*_*i*_	Model likelihood	K
Ψ_CELL_ (NDVI+TRI), θ^0^(.), θ^1^(.),γ (.),ε(.),p(season), θ^0^pi()	739.47	0.00	0.78	1.00	10
Ψ_CELL_ (NDVI), θ^0^(.), θ^1^(.),γ (.),ε(.),p(season), θ^0^pi()	742.04	2.57	0.21	0.28	9
Ψ_CELL_ (TRI), θ^0^(.), θ^1^(.),γ (.),ε(.),p(season), θ^0^pi()	748.20	8.73	0.01	0.01	9
Ψ_CELL_ (.),θ^0^(.), θ^1^(.),γ (.),ε(.),p(season), θ^0^pi()	767.99	28.52	0.00	0.00	8
Ψ_CELL_ (DNS), θ^0^(.), θ^1^(.),γ (.),ε(.),p(season), θ^0^pi()	768.58	29.11	0.00	0.00	9
Ψ_CELL_ (CC), θ^0^(.), θ^1^(.),γ (.),ε(.),p(season), θ^0^pi()	769.51	30.04	0.00	0.00	9
Ψ_CELL_ (HAB), θ^0^(.), θ^1^(.),γ (.),ε(.),p(season), θ^0^pi()	769.71	30.24	0.00	0.00	9
Ψ_CELL_(.),θ^0^(.), θ^1^(.),p(),θ^0^pi()	791.49	52.02	0.00	0.00	4

Ψ_CELL_ = probability of site occupancy/habitat use at the grid cell level; *p* = probability of detection; θ^0^ = spatial dependence parameter representing the probability that the species is present locally, given the species was not present in the previous spatial replicate; θ^1^ = spatial dependence parameter representing the probability that a species is present locally, given it was present at the previous spatial replicate. γ is the probability that the site is occupied in summer season, given that it was unoccupied in winter season. ε is the probability that the site is unoccupied in summer season, given that it was occupied in winter season. pi is the probability that initial replicate is preceded by an occupied site. HAB: habitat available; CC: canopy cover; NDVI: normalized difference vegetation index; TRI: terrain ruggedness index; DNS: distance to nearest settlement; *w*_*i*_: model weight; K: number of parameters.

**Table 5:** Summary of estimates of β coefficients from the logit link function based on the best and univariate model estimates for competing models within 2 delta AIC of the top model or containing a model weight more than 95%, based on landscape level covariates hypothesized to influence probability of habitat use at the 3.24 km^2^ grid cell level (Ψ_CELL_); Models in bold and underlined represent the best models and models in italics represent robust beta estimates (95% CI do not include zero).

Model	β_O_ (SE)	β_DNS_ (SE)	β_TRI_ (SE)	β_HAB_ (SE)	β_CC_ (SE)	β_NDVI_ (SE)
Best model			**0.92 (0.48)**			***1*.*80 (0*.*80****)*
Univariate	NA	-0.31 (0.26)	*1*.*68 (0*.*54)*	-0.32 (0.66)	0.20 (0.30)	*2*.*47(0*.*83)*

DNS: distance to nearest settlement; TRI: terrain ruggedness index; HAB: habitat available; CC: canopy cover; NDVI: normalized difference vegetation index; SE represents unconditional standard errors.

The model averaged estimate of probability of elephant habitat use ($\hat{\Psi }SE(\hat{\Psi })$) was 0.51 (0.02) in winter and 0.57 (0.02) in summer in the Churia habitat. The site-specific variation in habitat use by elephants shows that elephants intensively used certain habitat more than others across the seasons (Fig 5). The area of available habitat-used by elephants out of the total 537 km^2^ of potential available Churia habitat was 271 km^2^ and 302 km^2^ in winter and summer, respectively. The changes in habitat use between seasons (lambda, λ) showed a positive trend, $\hat{\lambda }$ (SE) = 1.04 (0.15), such that area used increased by 4% from the winter to summer season, but variability was high. We also found a higher colonization (i.e. became used) probability (γ, gamma, 0.21(0.02)) than extirpation (i.e. because unused) probability (ε, epsilon, 0.10(0.01)) of habitat use in summer across the Churia habitat.

## Discussion

This is the first systematic application of camera trapping and sign surveys using an occupancy framework to quantitatively assess elephant temporal activity, distribution, and habitat-use patterns in an ecologically fragile, yet important physiographic zone in Nepal. This study confirms that the often forgotten Churia habitat is ecologically important for elephants in addition to potentially 23 other mammalian species identified in photos [8]. Our study shows the first photographic images of elephants in Churia habitat and demonstrates that camera traps are not only valuable tools for carnivores like tigers, but also for species inventories and monitoring of certain mammal species, including the little-known, Asian forest elephants. Elephant detection followed by the records of a breeding population (with calves at two locations) within the Churia suggests that seasonally dry Churia habitat is also an ecologically important habitat for large-sized ungulates. Asian elephants use multiple forest habitats ranging from semi-arid, dry thorn to wet evergreen forest, and they attain highest densities in the moist and dry deciduous forests containing substantial grass and bamboo forage [40, 41]. Moist and dry deciduous forest in Churia habitat harbors a dense under growth along with large fragments of bamboo providing potential forage for an elephant population. Bamboo distribution within lowland forest is rare, thus the presence of bamboo habitat could cause elephant to be attracted to Churia habitat.

We found elephant activity patterns in winter to be mainly nocturnal, with crepuscular peaks. Elephant activity recorded in Churia habitat was similar to forest elephants in Africa [42] and Asia [43]. The temperatures in our study area rose as high as 32°C during the day even in winter, which may result in nocturnal activity to avoid heat during daytime. Environmental temperatures have been shown to dictate elephant activity within a day in other studies, with potential consequences for fine-scale habitat selection, space use, and foraging [44].

Recent advancements in occupancy models have allowed ecologists to model detection/non-detection data while accounting for imperfect detection [15] and incorporate spatial auto-correlation. Sign surveys provide a high-quality snapshot of elephant use of forests with high probabilities of detection across seasons. Overall results show that elephants exhibit site specific variation in habitat use along the Churia habitat. We found large variation in the habitat use in two sections of Churia (east versus and west, Fig 5) with the eastern section having a much higher probability of habitat use at 67% than the western section at 24%. This likely occurs because the eastern section of the Churia, which is contiguous with Parsa National Park, provides more contiguous habitat that is potentially more likely to support the large ranging behavior of elephants [45] in the central landscape. Moreover, habitat features favored by elephants (i.e. covariates NDVI and TRI that increased probability of habitat use) are more widespread in the eastern section than in the western part of Churia habitat.

Elephants often use closed forest to seek shade from the sun and heat during the hottest hours of the day likely resulting in more clumped distribution in summer than in the winter (where distribution is more spread out)[46]. Substrate conditions of Churia are dynamic as degree of fragility increases with the progression into the summer season, causing difficulties in detecting signs. This could be one of the reasons for lower probability in detecting elephant sign in Churia habitat during summer season. The difference in probability of habitat use between summer (high) and winter (low) suggest seasonality in elephant habitat use. Forest fires occur quite late in the Churia compared to the adjacent lowland areas and forest undergrowth in Churia provides forage for forest ungulates including elephants. Elephant protein requirements are higher in summer, yet forage availability in the lowlands is relatively low, while in Churia, herb and grass availability is relatively high, thus Churia habitat might be sought out by elephants in summer. These seasonally driven vegetation dynamics perhaps dictate elephant responses to habitat changes, especially in the summer dry seasons, causing them to move to more suitable sites within the Churia as the lowland fires clear forage earlier in the season [47]. Although detection probability was lower in summer, habitat use was higher (~4% increase), likely due to migration of more elephants from adjoining Churia habitat in Parsa National Park.

The environmental covariate of NDVI (cumulative AIC weight: 98%) showed strong support for positive effects of productivity on elephant use of Churia habitat. Our finding is consistent with the suggestion made by Lakshminaryanan et al. [24] that probability of elephant habitat use is associated with NDVI. Sites with higher NDVI values are indicative of moist forests that result in higher probability of habitat use, thus resulting in site specific variation in habitat use in Churia. Kamala trees (*Mallotus philippinnesis)*, date palm *(Phoenix dactylifera*), and bamboo (*Bambusa vulgaris*) are found extensively around Churia hills and are among the preferred fodder of elephants [48, 49] in the region. Ground variables such as density of these important plant species could further refine and better predict habitat use patterns within undulating Churia habitat. We found weak support for preference of more rugged sites (TRI) in determining the site-specific variation in elephant habitat use. Our result with TRI was opposite to our apriori. Presence of forage and water availability along Churia could be driving elephant presence even in areas of high ruggedness.

## Conclusions

Our results highlight the global conservation value for elephants of the seasonally dry, deciduous forests of the Churia hills in Chitwan National Park and the Siwaliks range across Nepal and India. Elephants were widely distributed across the Churia and they displayed high use of the eastern portion of the habitat in both seasons, and increased use of the western portion in summer. We also found site-specific variation in habitat use dictated by preference for higher vegetation productivity and slight but variable preference for higher elevation. Thus, habitat use across the heterogenous landscape is not random but depends upon site specific characteristics. Churia habitat faces a myriad of problems including large linear infrastructure development passing through the habitat. Churia habitat within the national park is well protected, yet still faces challenges outside of parks with respect to deforestation, forest fires, and encroachment leading to human-elephant conflict. Among endangered species, the elephant is a mega herbivore and could serve as potential indicator species for measuring effectiveness of Churia conservation within high priority areas such as protected areas, corridors, and community forests. The occupancy framework in this study also permits evaluation of management interventions across the landscape [24]. The Government of Nepal’s flagship monitoring program using camera trapping and occupancy surveys is conducted every five years across this landscape and can now specifically include elephants for the purpose of determining occupancy and habitat use dynamics over time [16]. Government investment in landscape wide camera trap surveys, with an average effort of 27,829 camera trap nights in monitoring terrestrial animals, can also include elephants as “target species” and Churia as “target habitat” for long term monitoring.

Our study did not estimate density (number of elephants per 100 km^2^) as individual elephants were difficult to distinguish and the survey design was more suited towards terrestrial carnivores (body size~ 200kg; shoulder height~0.3–0.6 m). Future designs specific to elephants, combined with spatially explicit capture-recapture (SECR) framework, could offer an opportunity to estimate elephant density using morphological traits to identify individuals [50].

Kanagaraj et al.[51] suggested that a mixture of poor and high quality habitat can provide connectivity to core areas across fragmented habitat in the region, and Churia habitat could offer such important connection and should be targeted in elephant conservation plans. Thus, we should not forget the seasonally dry Churia habitat, but rather include it as ecologically important for long-term persistence of elephants and other forest ungulates across the landscape.

## Supporting information

S1 Table Grid and season wise elephant probability of habitat use estimates. (XLSX)
